# Supplementary material for: A Network Landscape of HPVOPC Reveals Methylation Alterations as Significant Drivers of Gene Expression via an Immune-Mediated GPCR Signal
Source: Cancers (Basel). 2023 Sep 1;15(17):4379. doi: 10.3390/cancers15174379 (PMC10486378; doi:10.3390/cancers15174379)
Supplement: Supplementary file 1 [file cancers-15-04379-s001.zip › cancers-2533497-supplementary.pdf]

Suppl. Figure S1

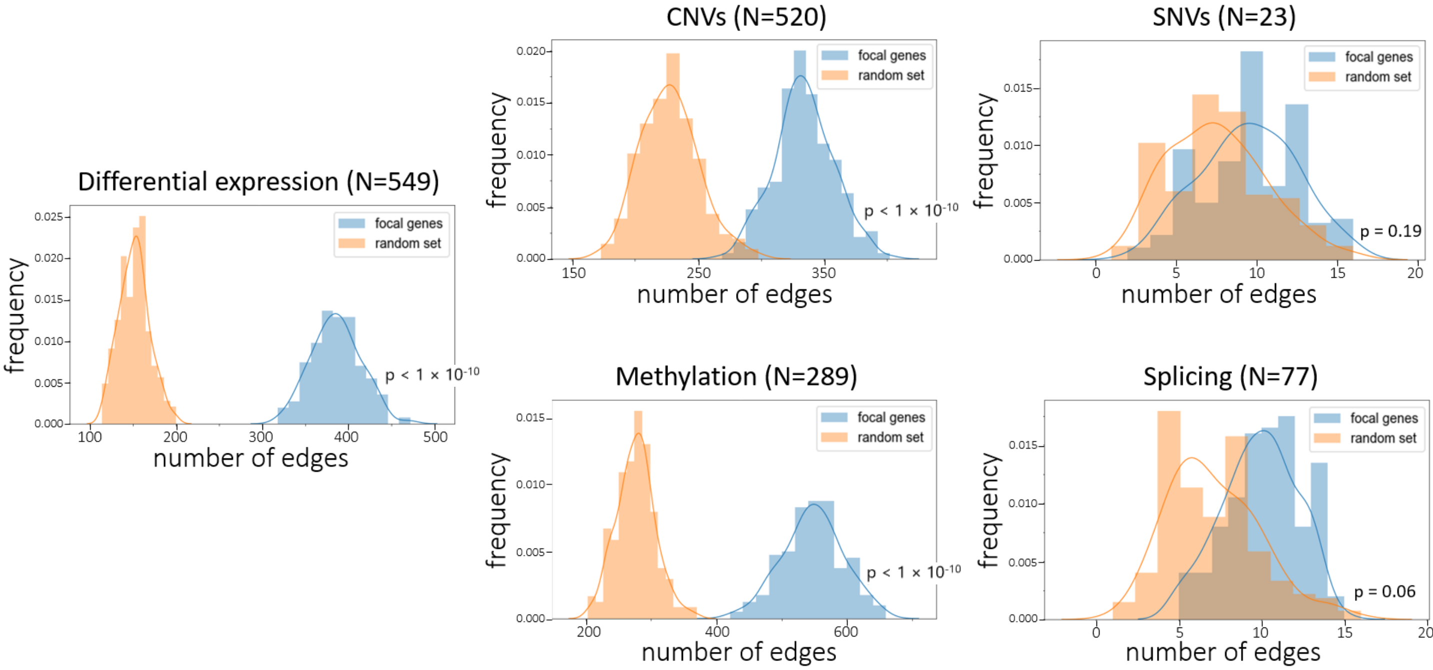

Supplemental Figure S1. Differential expression, CNV, and methylation network analysis gene sets are significantly more connected than degree-matched random sets.

Edge localization histograms for each tumor alteration class following network analysis demonstrate significantly more connected sets for differential expression, CNV and methylation networks. Shown in blue are focal genes for each alteration class and in orange a degree-matched random gene set for statistical comparison. Note that this method has more power to detect significance with larger gene sets, because small gene sets have on average very few edges connecting their nodes in the interactome. [CNV = copy number variation, SNV = single nucleotide variation]

Suppl. Figure S2

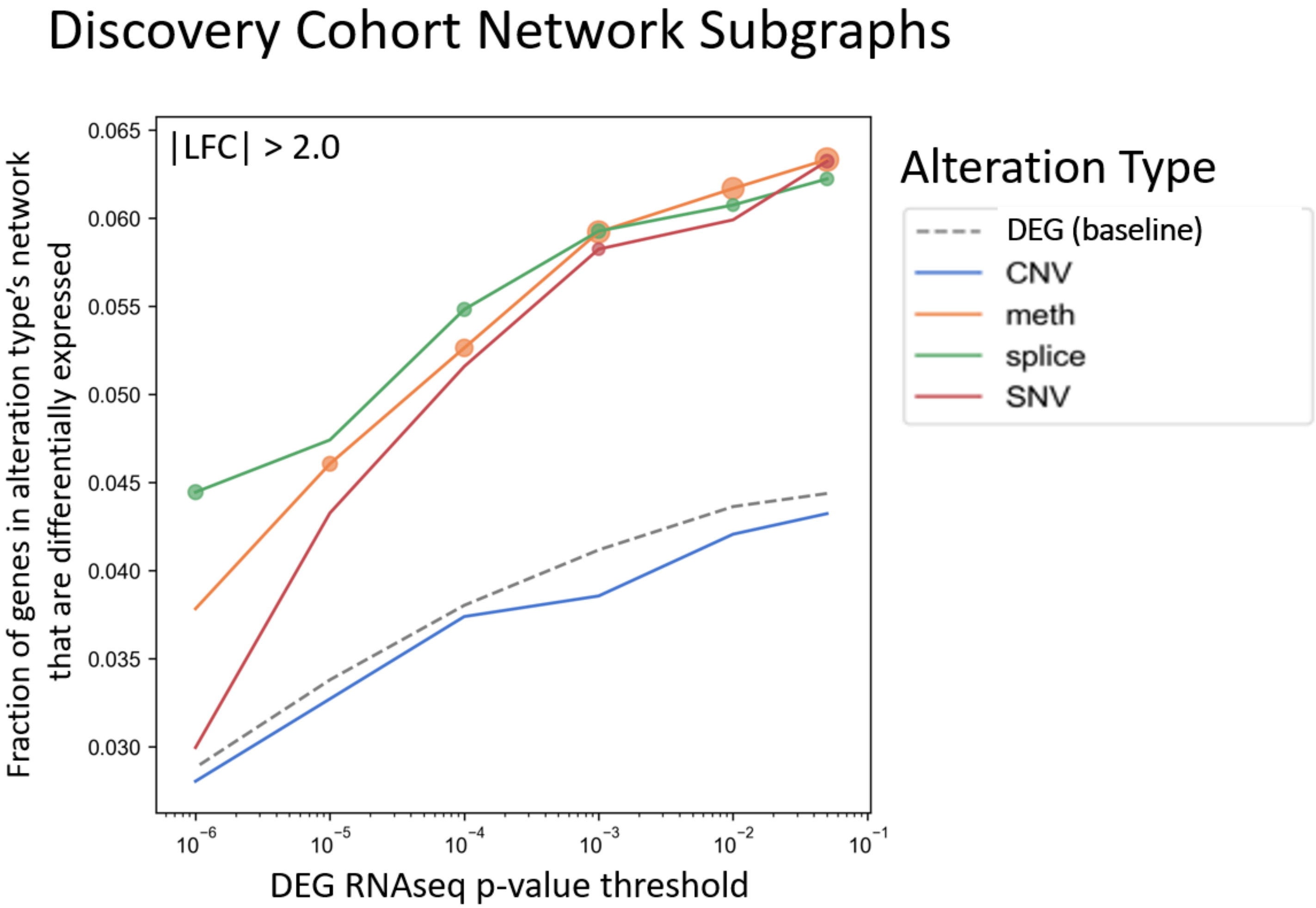

Supplemental Figure S2. Splicing, methylation, and SNV alteration networks are enriched for differentially expressed genes (magnitude LFC > 2)

To visualize the enrichment for differentially expressed genes within each tumor alteration type after network propagation analysis, the fraction of genes for each alteration type's network subgraph that are differentially expressed (LFC magnitude of 2 or greater) was plotted against varying p-value significance thresholds for differential expression (RNAseq) for the discovery cohort. Bubble size is proportional to increasing statistical significance of enrichment for DEGs among each alteration type's network subgraph. [DEG = Differentially expressed genes, METH = methylation, SPLICE = Alternative splice events, CNV = copy number variation, SNV = single nucleotide variation, LFC = log-fold change in gene expression]

Suppl. Figure S3

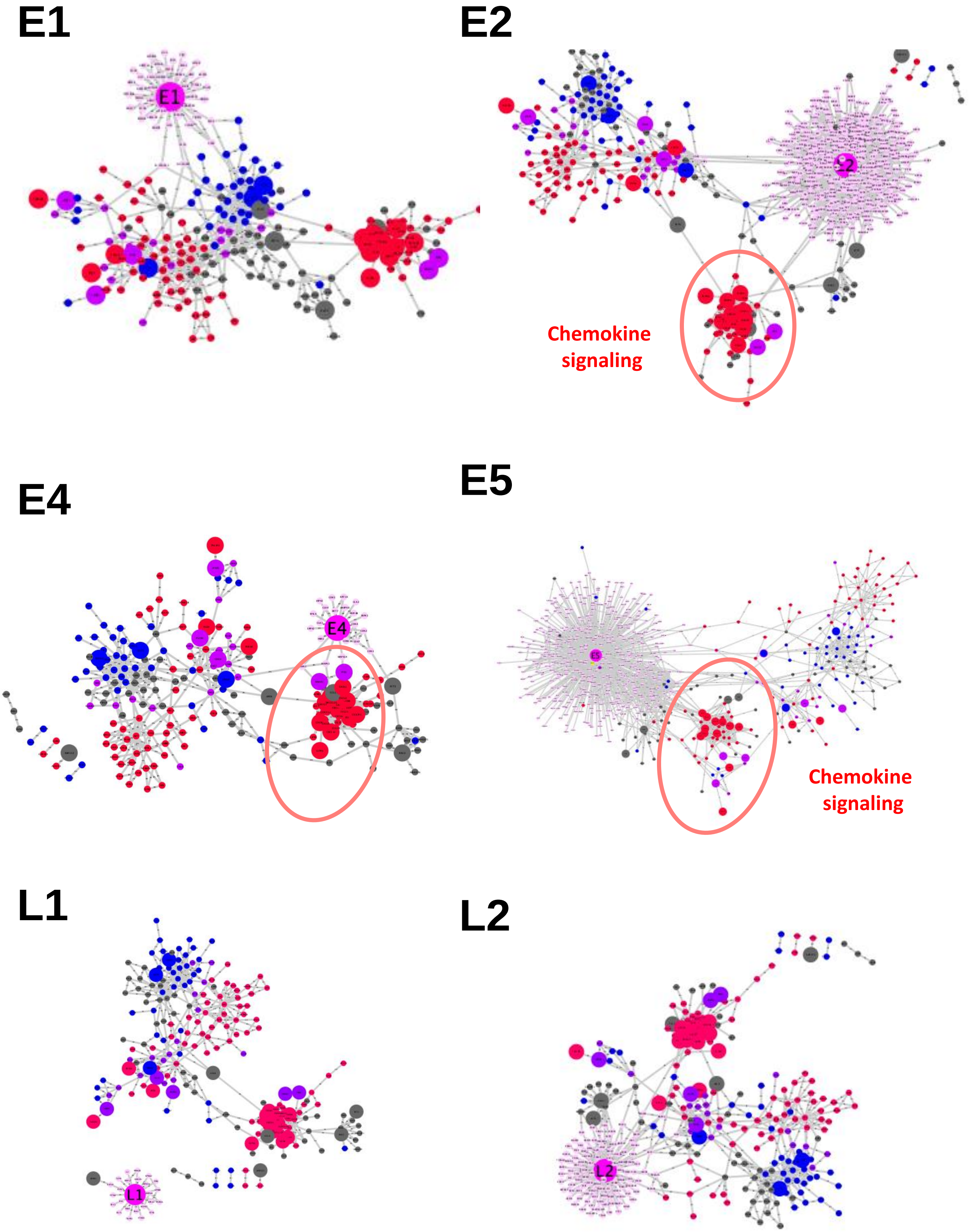

Supplemental Figure S3. GPCR signaling protein interaction with HPV16 viral proteins demonstrates significant interaction between E2/E4/E5 and chemokine signaling proteins.

Cytoscape visualizations of publicly available data of interactions between HPV16 viral proteins E1, E2, E4, E5, L1, and L2 (**E6 and E7 are shown in Figure 4**) and known human GPCR signaling proteins (red dots for proteins annotated in reactome as G alpha (i), blue dots for G alpha (q), and purple dots proteins annotated as both). Red circles emphasize remarkable interaction between viral proteins E2/E4/E5 and chemokine signaling proteins.

Suppl. Figure S4

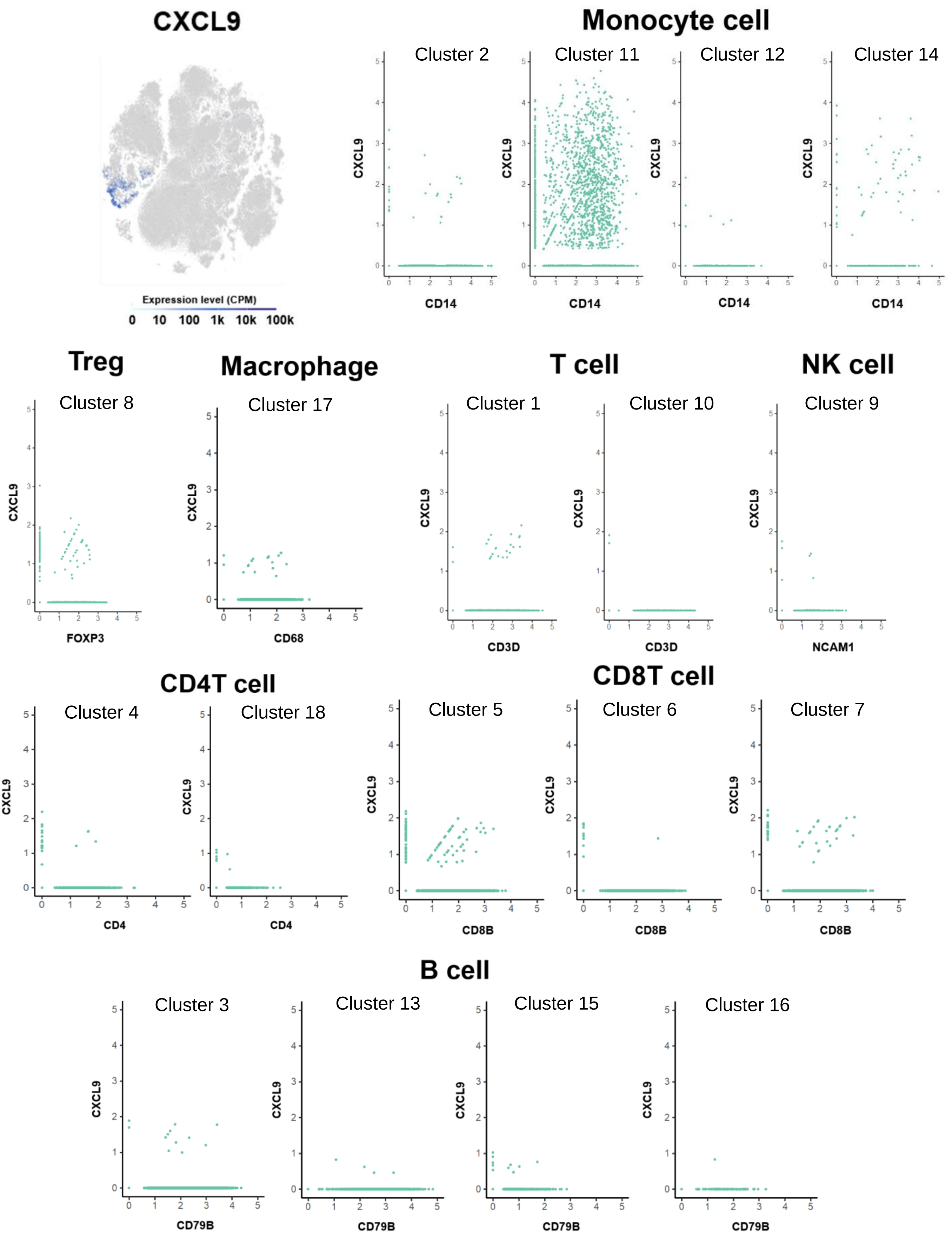

Supplemental Figure S4. *CXCL-9* expression among immune cell populations in HPVOPC shows highest expression by CD14+ Monocytes.

t-SNE plot of publicly available scRNA-seq HPV-related OPC data showing the distribution of *CXCL9* expression and plots of *CXCL9* expression among various immune cell types.

Suppl. Figure S5

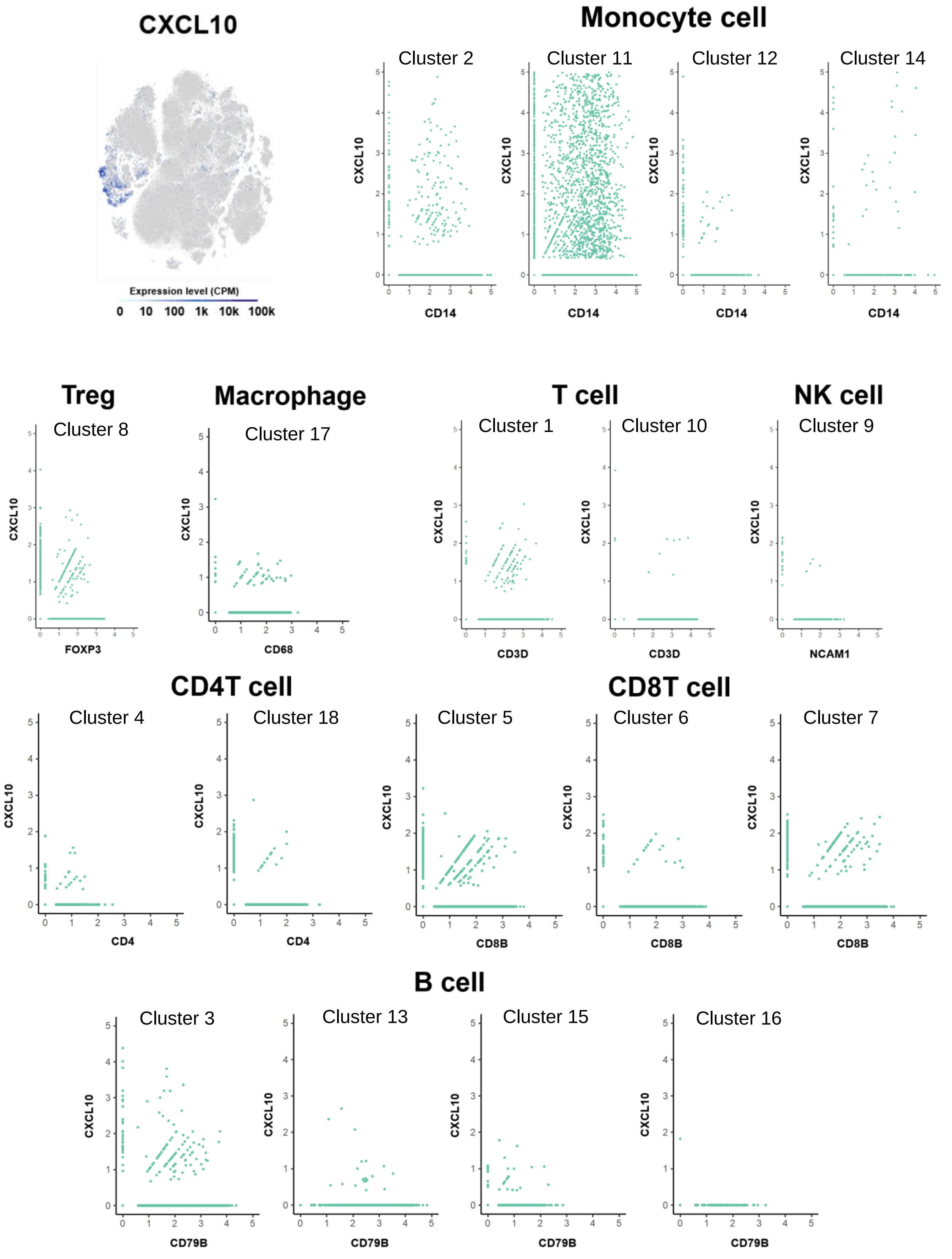

Supplemental Figure S5. *CXCL-10* expression among immune cell populations in HPVOPC shows highest expression by CD14+ Monocytes.

t-SNE plot of publicly available scRNA-seq HPVOPC data showing the distribution of *CXCL10* expression and plots of *CXCL10* expression among various immune cell types.

Suppl. Figure S6

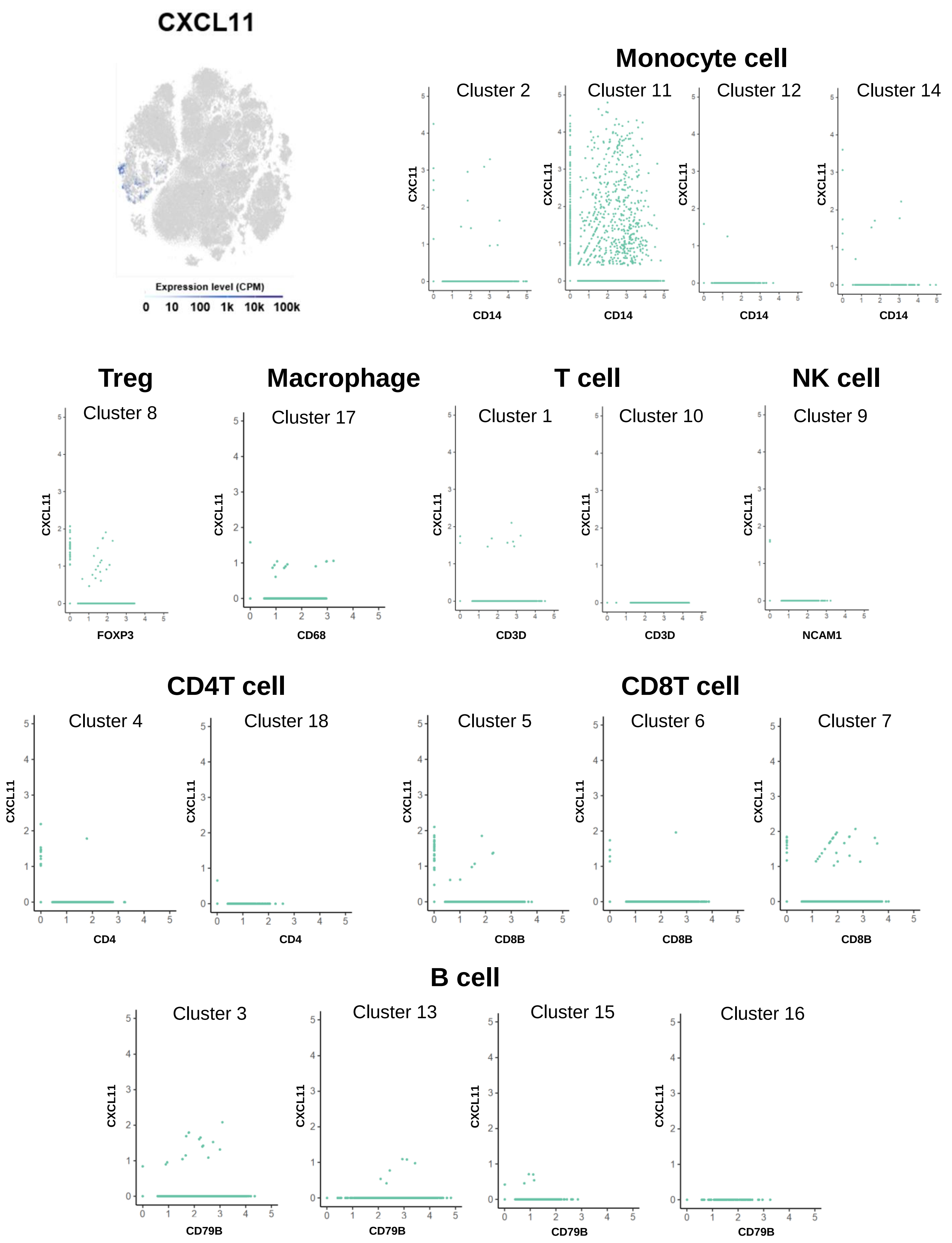

Supplemental Figure S6. *CXCL-11* expression among immune cell populations in HPVOPC shows highest expression by CD14+ Monocytes.

t-SNE plot of publicly available scRNA-seq HPVOPC data showing the distribution of *CXCL11* expression and plots of *CXCL11* expression among various immune cell types.
